# Plant latent defense response against compatibility

**DOI:** 10.1038/s41396-023-01399-9

**Published:** 2023-03-29

**Authors:** Huiming Zhang

**Affiliations:** Shanghai Center for Plant Stress Biology, Center for Excellence in Molecular Plant Sciences, Chinese Academy of Sciences, Shanghai, 201602 China

**Keywords:** Plant sciences, Bacterial host response

## Abstract

Managing the association with microbes is crucial for plants. Evidence is emerging for the plant latent defense response, which is conditionally elicited by certain microbial nonpathogenic factors and thereby guards against potential risks from beneficial or commensal microbes. Latent defense response is an exciting new research area with a number of key issues immediately awaiting exploration. A detailed understanding of latent defense response will underpin the applications of beneficial microbes.

Plants naturally live with a wide variety of soil microbes, which can be roughly categorized as pathogenic, commensal, or beneficial based on the microbial impacts on their associated plants, although the impacts caused by the same microbe can be either generalized or plant species-specific while the outcome from a given binary relation is subject to influences by other factors in the complex environment. Unlike commensal microbes that impose neither benefits nor harms to the plants, beneficial microbes promote plant growth or improve plant tolerance to certain stress conditions, whereas pathogens impose threats that in some cases can be deadly to plants [1]. Hence, the detection of and responses to microbial factors are critical for plant survival.

In response to pathogens, the plant immune system is typically activated through microbe-associated molecular pattern (MAMP)-triggered immunity (MTI) and effector-triggered immunity (ETI) [2]. The perception of MAMPs, such as the bacterial flagella epitope flg22, by cell surface-localized pattern recognition receptors (PRRs) initiates MTI without inducing plant cell death. When confronting pathogens that have evolved abilities to subvert MTI through effector proteins released to the apoplast or into the host cells, plants use the evolved nucleotide-binding oligomerization domain-like receptors (NLRs) to detect the effectors directly or indirectly, resulting in the formation of oligomeric sensor complexes that trigger ETI and oftentimes cell death [3, 4]. Plant PRRs also perceives damage-associated molecular patterns (DAMPs), which are plant-derived molecules resulting from cell wall damages and can potentiate MTI [5]. In addition to local defense responses, plants infected by pathogens can produce mobile signals that travel from the infected tissue to distal tissues, resulting in systemic acquired resistance (SAR) that helps protect the plant from future infections [6].

MAMPs are immunogenic signals produced by not only pathogens but also beneficial and commensal microbes [7]. Detection of these extracellular danger signals not only allows for timely defense against pathogens, but also presents obstacles to the establishment of mutualistic association with beneficial microbes. To achieve successful association with plants, microbes have evolved various strategies to avoid or subvert MTI. The evasion of MAMPs from PRR recognition results from alterations in the immunogenic sequences, meanwhile, microbial suppression of MTI can involve extracellular factors that suppress the production of MAMPs, in addition to the intracellular effectors that subvert plant immune signaling [8, 9]. As a result of the suppression of immunity, plant compatibility with pathogens leads to disease, whereas the compatibility with beneficial microbes underlies mutualism. However, in contrast to showing hostility to pathogens, plants show amity with beneficial microbes, because the compatible associations with beneficial microbes are also contributed by plants, as known in rhizobia-legume symbiosis and plant symbiosis with arbuscular mycorrhizal (AM) fungi. Nitrogen-deficient legumes secrete flavonoids that trigger rhizobial synthesis of nodulation factors (Nod factors), which in turn elicit plant signaling that provokes the deformation of root hairs and the formation of nodule primordium [10, 11]; similarly, plants attract AM fungi by root secretion of strigolactones, which trigger extensive hyphal branching and are induced by plant phosphorus deficiency and potentially by nitrogen deficiency in a way that is plant species-specific [12, 13]. The plant’s initiative in establishing mutualism is further supported by plant suppression of MTI, as exemplified by several *Medicago truncatula* genes that function sequentially in suppressing plant defense against the rhizobial symbionts in nodules [14], as well as by the rice symbiotic receptor OsMYR1 that suppresses OsCERK1-mediated MTI for establishing mutualism with AM fungi [15]. In addition, in the differentiation zone of *Arabidopsis thaliana* roots, MTI is normally limited due to low expression levels of PRRs but is strongly mounted by local cell damages, thereby providing a way to respond appropriately to pathogens while sparing beneficial and commensal microbes [5].

Although the plant-microbe mutualism is co-established by both partners, evidence is accumulating that the mutualism may be conditionally switched to incompatibility, suggesting that plants somehow are capable of controlling the compatibility with beneficial microbes. Microbes produce and release a complex array of metabolites that can potentially be perceived by neighboring plants [16]. Unlike MAMPs that are perceived by plants as danger signals [2, 7], many microbial extracellular metabolites are nonpathogenic factors (NPFs) that seemingly do not elicit plant defense responses, and as such, NPFs appear negligible regarding compatibility.

Recent studies showed that plants are capable of controlling the compatibility with beneficial bacteria through the perception of certain NPFs. *Bacillus amyloliquefaciens* strain GB03 is a plant-beneficial bacterium capable of triggering plant growth-promotion (PGP) through soil inoculation or volatile emissions [17]. The PGP induced by GB03 volatile emissions results from integrated plant responses to the different volatile components. While it remains unclear how the volatile signals initiate plant cellular responses, the known downstream mechanisms include modulation of auxin and abscisic acid signaling [18, 19], enhancement of iron and sulfur assimilation [20, 21], enhancement of photosynthetic apparatus production [19], protection of chlorophylls from stress-induced degradation [22], and the requirement of AGP19 that is important for cell division and expansion [23]. The Arabidopsis plants defected in salicylic acid accumulation (*nahG*) or signaling (*eds1*) showed similar fresh weights as the wild type plants [24], indicating that decreasing the basal immunity is not sufficient to cause PGP; in contrast, several NPFs in the GB03 volatile emissions strongly elicited plant immunity under certain conditions and compromised the PGP, showing not only the trade-off between growth and immunity, but also the antagonism against compatibility as evidenced by the immunity-dependent impairment of bacterial colonization [24, 25].

In phosphate (Pi)-sufficient *A. thaliana*, the bacterial volatile component diacetyl emitted from GB03 suppressed plant immunity in supporting the mutualistic association, whereas in Pi-deficient plants, diacetyl strongly induced plant defense that was mediated by salicylic acid [25]. Phosphorus is an essential macronutrient for both plants and bacteria. Under Pi-deficient conditions, activating immunity could be an optimized strategy for plant Pi acquisition because by doing so, plants can deter bacteria from root colonization and thereby reduce direct competition for Pi; meanwhile, the plants can still take advantage of the bacteria within the rhizosphere if the bacteria are capable of Pi solubilization or producing other plant-beneficial traits. Therefore, the differential responses to diacetyl under different Pi availability suggest a plant’s initiative in controlling compatibility with beneficial microbes through the perception of certain NPFs [25–27].

Plant regulation of the compatibility with beneficial microbes through the perception of NPFs was also observed in the Arabidopsis mutant *rol1* (*regulator of LDR 1*), which was isolated from a forward genetic screen for mutants with defective plant growth promotion triggered by *B. amyloliquefaciens* GB03 [24]. In wild-type plants, GB03 enhanced chloroplastic lipid biosynthesis that consumed oleic acid. In the *rol1* mutant, biosynthesis of oleic acid was impaired, resulting in severe disruption in the plant lipidome that was exacerbated by GB03. This exacerbation indicates that GB03 increases the vulnerability of *rol1* to potential threats since fatty acids and lipids are important and often essential for various cellular functions [28]. Concomitantly, the *rol1* mutant plants responded to GB03 with strong activation of defense, which was mediated through the plant perception of the NPFs 2,3-butanediol, acetoin and 2-methyl-1-propanol in the volatile emissions from GB03.

This hidden layer of plant defense highlights the importance of NPFs in mediating plant regulation of mutualism, leading to the concept of the latent defense response (LDR), which is conditionally activated by certain NPFs and antagonizes compatibility [24]. LDR indicates the plant’s initiative in determining the plant-microbe association for optimized benefits, because the conditional activation of LDR avoids unnecessary hostility to beneficial or commensal microbes while enabling plants to deter the same microbes when the microbial association is unfavorable to the plants. The functional importance of LDR is also because it provides a safeguard mechanism for antagonizing mutualism and commensalism, in which MTI is already absent or suppressed in the binary relations. Although the functional importance of LDR is especially highlighted in mutualism and commensalism, it should be noted that LDR would also antagonize the passive compatibility with pathogens that successfully develop diseases. Given that the association between plants and beneficial microbes is as common as plant interactions with pathogens, the plant’s ability of perceiving certain NPFs for controlling mutualism is as important as the ability of perceiving MAMPs for avoiding pathogenicity, i.e., in parallel to MAMP-triggered immunity that promptly defends against pathogens, NPF-triggered LDR protects against potential risks from beneficial or commensal microbes (Fig. 1).

**Fig. 1:**
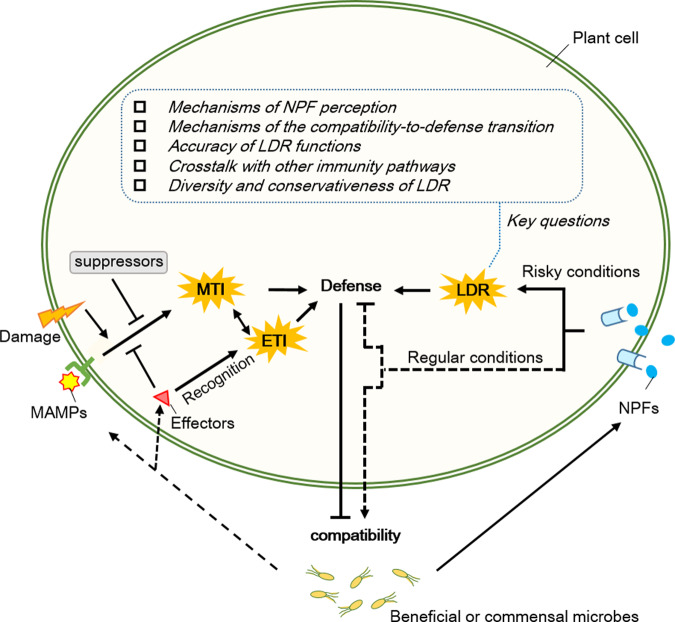
A perspective on the plant latent defense response (LDR). Between plants and the associated beneficial or commensal microbes, the compatibility is co-established by the microbial and plant suppression of MTI. Host-specific recognition of the effectors or defectiveness in the plant MTI suppressors leads to defense against the compatibility. While showing amity to beneficial and commensal microbes, plants deploy LDR as a safeguard mechanism for antagonizing the mutualism and commensalism, in which MTI is absent or suppressed for the compatibility. Activation of LDR is triggered by certain NPFs and occurs only under risky conditions to the plant. The conditional activation of LDR avoids unnecessary hostility to beneficial and commensal microbes while enabling plants to deter the microbial association when it is unfavorable. The new research field of LDR awaits exploration. MAMP microbe-associated molecular pattern, MTI MAMP-triggered immunity, ETI effector-triggered immunity, NPF non-pathogenic factor. The dashed lines pointing to MAMPs and effectors indicate that some compatible microbes may evade immune detection. The dashed lines from NPFs indicate that some NPFs may contribute to compatibility under normal conditions, such as diacetyl that suppresses some immune responses.

The phenomena of plants showing incompatibility with beneficial microbes are accumulating. In particular, legumes and rhizobia form symbiotic relations but incompatibility can occur, often resulting from either failures in MTI suppression or plant ecotype-specific recognition of bacterial effectors that elicit ETI [14]. These types of incompatibility should not be confused with the incompatibility caused by LDR, because the former are caused by danger signals whereas the latter are activated by NPFs only under conditions that are unwelcomed by the plants. Interestingly, mutations in *M. truncatula* DNF2, a putative phosphatidylinositol phospholipase C-like protein, led to defense reactions and rapid senescence in the nodule when the plants were grown with agar as the medium-solidifying agent; in contrast, the disruption of rhizobial symbiosis in *dnf2* mutants was not observed when the agar was replaced by agarose, which is purified agar containing no agaropectins and harboring reduced levels of unidentified impurities [29]. Agar is not known to elicit or prime plant defense, meanwhile, adding flg22 or chitin to the agarose medium did not mimic the effects of the agar [29]. Thus, the conditional activation of defense in *dnf2* mutants is reminiscent to LDR, albeit the defense elicitor and the underlying mechanism remain unclear.

LDR is characterized by the conditional activation, in which nutrient availability can be a key determinant. In *A. thaliana*, the transcriptional regulator PHR1 not only positively regulates phosphate starvation responses (PSR), but also negatively regulates plant defense through promoter occupancy at its target genes [30]. Under Pi deficient conditions, the recruitment of PHR1 to PSR gene loci likely would decrease the promoter occupancy by PHR1 at the defense-related genes, since PHR1 gene expression is only weakly responsive to Pi starvation [31]. In such a scenario, Pi deficiency would create a permissive environment for transcriptional activities at the PHR1-suppressed defense-related genes, thereby allowing for diacetyl-mediated elicitation of LDR. Pi deficiency also impairs the symbiosis between legumes and the nitrogen (N)-fixing rhizobia. As shown in common bean (*Phaseolus vulgaris*), the reduction of nodule numbers under Pi deficiency was mediated through PvPHR1-dependent activation of the autoregulation of nodulation (AON) pathway, which controls nodule numbers for optimal balance between the costs for and the benefits from the mutualism with rhizobia [32, 33]. As shown in soybean (*Glycine max*), the nodule autoregulation receptor kinase (GmNARK) potentially links AON with defense [34]. It is unclear whether PHR1 in legume species play dual and opposite roles in regulating PSR and defense as in *A. thaliana*.

In some cases, the conditional activation of plant defense can also result from nutrient repleteness. For instance, the compatible association between *A. thaliana* with *Colletotrichum tofieldiae*, an endophytic fungus that can transfer Pi to the plant, occured only under Pi deficient conditions; whereas under Pi sufficient conditions, plants detered fungal colonization through the accumulation of antifungal metabolites [35]. Similarly, high Pi availability suppressed plant symbiosis with AM fungi, which facilitate Pi acquisition in approximately 80% of land plants; the suppression, as shown in rice, was due to the repression of OsPHR2 that governed the symbiosis gene regulatory network [36, 37]. It is interesting but unclear whether defense mechanisms contribute to the suppression of plant symbiosis with AM fungi under high Pi availability. Similar to Pi suppression of plant-fungi symbiosis, the symbiosis between legumes and rhizobia can be inhibited by N fertilizers especially nitrate [32, 38]. The negative regulatory pathway induced by nitrate converges with the AON pathway and affects each step of nodulation [32]. In a split-root study of *M. truncatula* symbiosis with *Sinorhizobium medicae*, mature symbiotic nodules responded to whole-plant N-satiety signaling with nodule senescence and defense activation [39]. Nitrate stimulated *M. truncatula* nodule production of nitric oxide (NO) that provoked early nodule senescence [40]. NO is an important mediator of plant defense, thus it may play a role in connecting immunity and senescence in the nodule, where the relationship between the two processes remained obscure. During symbiotic interactions, rhizobial Nod factor repressed and induced the production of reactive oxygen species at early and later stages, respectively, as exemplified in *M. truncatula* roots treated with *Sinorhizobium meliloti* Nod factor [41, 42]. Both induction and repression of defense-related gene expression were observed in Nod factor-treated *M. truncatula* root epidermis [43]. The ability of inducing host defense responses makes Nod factors somewhat reminiscent to NPFs that are capable of inducing LDR, since Nod factors induce nodule formation and are therefore commonly not considered as danger signals. It is intriguing whether nitrate-replete legume roots mount persistent defense in response to Nod factors. The nutrient repleteness-induced defense showed target specificity, in that the targeted microbes carried functions tightly related to the nutrients and were no longer needed for the plants, thereby pointing to the involvement of the yet-to-be identified microbial factors in the conditional activation of defense.

LDR is an exciting new research area with a number of key issues immediately awaiting exploration (Fig. 1). For each LDR event, the sensing of NPF signals and the signal transition that switches the compatibility to defense are the two core processes to be disclosed. In addition, it is an interesting question for each LDR event whether the function of the LDR is accurate or indiscriminate in repelling the plant-associated microbes that impose the risk to different degrees. Mutualistic relations between plants and microbes are commonly investigated as pairwise interactions, however, plants in natural environments are inevitably interacting with microbial communities consisting of different species in the same or different niches. Once LDR is activated by a particular microbial partner, how will the plant reshape the microbiome and rebalance the complex interactive network of all the plant-microbe interactions for optimal benefits? Particularly, it is intriguing whether LDR is less prone to be elicited in species-specific mutualism than generalist mutualism, since the former is an obligate relation whereas the latter may be reconstituted by other microbial partners.

Some pathogens may also produce NPFs capable of activating LDR, which can then reinforce plant disease resistance in addition to the contribution by the forefront MAMP-triggered immunity. Because NPFs do not behave like MAMPs in directly activating plant immunity, it is unlikely that NPFs are perceived through the same mechanisms as MAMPs. However, the plant immunity network functions as a concerted system such that different segments are inherently connected and coordinated, as demonstrated by the mutual potentiation between the MTI and ETI pathways [44, 45]. It is an important question as how LDR is connected with and coordinate with the other segments of the immunity network. A clear answer to this question would be built on knowledge about how plants perceive NPF signals, in addition to genetic dissections of the defense outputs from the plant-microbe interactions. Similarly, how different LDR pathways may interact with each other is also to be explored. For instance, the diacetyl-triggered LDR is mediated through a mechanism different from the LDR in *rol1*, because the latter is not induced by diacetyl [24, 25]. Moreover, the activation of LDR by its eliciting NPFs may be interfered indirectly by the plant developmental regulation or environmental responses, as well as by other factors from the NPF-producing microbes or the other microbes present in the same plant-associated community.

The diversity of LDR mechanisms and the conservativeness of LDR phenomena are two other key issues awaiting to be addressed. On one hand, there are numerous species and combinations of beneficial microbes and their host organisms in nature, so species-based diversity in LDR mechanisms can be expected. For plants, Pi deficiency and oleic acid deficiency are not the only two threats that can be affected by microbes; thus, the diversity of LDR mechanisms likely would also come from different high-risk conditions that allow for LDR activation. In addition, while beneficial microbes may help plants combat environmental stressors, the compatibility may be abandoned by plants when it becomes unnecessary. On the other hand, environmental stressors, such as Pi deficiency, are common threats to different plant species and thereby may commonly influence plant relations with commensal or beneficial microbes. This commonality may confer conservativeness of LDR mechanisms across different plant species. The conservativeness of certain LDR mechanisms is likely also contributed by the fact that the same NPF can be produced by different microbes. A detailed understanding of these and other questions in the field of LDR will underpin the applications of beneficial microbes.
